# Occurrence of co-existing *bla*_VIM-2_ and *bla*_NDM-1_ in clinical isolates of *Pseudomonas aeruginosa* from India

**DOI:** 10.1186/s12941-016-0146-0

**Published:** 2016-05-06

**Authors:** Deepjyoti Paul, Debadatta Dhar, Anand Prakash Maurya, Shweta Mishra, Gauri Dutt Sharma, Atanu Chakravarty, Amitabha Bhattacharjee

**Affiliations:** Department of Microbiology, Assam University, Silchar, India; Department of Microbiology, Silchar Medical College and Hospital, Silchar, India; Department of Microbiology, Institute of Medical Sciences, Banaras Hindu University, Varanasi, India; Department of Life Science and Bioinformatics, Assam University, Silchar, India

**Keywords:** *bla*_VIM-2_, *bla*_NDM-1_, Integron, Inc F type, *Pseudomonas aeruginosa*

## Abstract

**Background:**

*bla*_VIM-2_ harboring *Pseudomonas aeruginosa* has been reported worldwide and considered as the most prevalent metallo-β-lactamase after NDM which are found horizontally transferable and mostly associated with integron gene cassettes. The present study investigates the genetic background, transmission dynamics as well as stability of *bla*_VIM-2_ in clinical isolates of *P. aeruginosa* harbor *bla*_NDM-1_ as well which were collected from October 2012 to September 2013.

**Methods:**

Two *P. aeruginosa* strains harboring *bla*_VIM-2_ along with *bla*_NDM-1_ were isolated from Silchar Medical College and Hospital, India. Genetic environment of these resistance determinants was determined and transferability was checked by transformation and conjugation assay which was further confirmed by Southern hybridization. Replicon typing was performed to determine the incompatibility group of the resistant plasmid and their stability was checked by serial passage method. Antimicrobial susceptibility pattern of the isolates was determined and their clonal relatedness was checked by pulsed field gel electrophoresis.

**Results:**

*bla*_VIM-2_ was found to be horizontally transferable through an Inc F type plasmid of approximately 30 kb in size. *bla*_VIM-2_ was found to be associated with integron gene cassette and was flanked by two different types of cassette arrays. Both the isolates were co-harboring *bla*_NDM-1_ which was carried within Inc N type of plasmid with an approximate 24 kb in size and associated with IS*Aba125* in their upstream region. Reduced susceptibility rate as well as high MIC range was observed in case of wild strains and transformants carrying *bla*_VIM-2_ and *bla*_NDM-1_.

**Conclusions:**

The detection of this co-existence of multiple carbapenem resistance genes in this part of world is worrisome and further investigation is required in order to trace the source and to initiate proper treatment option.

## Background

Carbapenems are considered as the last drug of choice for most of the serious infections caused by Gram negative bacteria, but due to the prevalence of multidrug resistant organisms these lifesaving drugs were compromised in treating the patients with severe illness. Gram negative bacteria have been documented as the most serious threat in the management of acute infections, as of exhibiting resistance to antibiotics due to the production of carbapenemase enzyme especially metallo-β-lactamase [1, 2]. *Pseudomonas aeruginosa* is one of the most common nosocomial pathogen causing acute infections and frequently reported for carbapenem resistance. VIM metallo-β-lactamase, the second most predominant MBL type responsible for antimicrobial resistance, is pandemic and becoming a health hazard. Among the 37 variant of VIM enzymes, *bla*_VIM-2_ has now been spread as prevalent MBL among *P. aeruginosa* in all European countries [3] whereas *bla*_NDM-1_ and *bla*_VIM-18_ has known to be originated from India [4, 5] where irrational use of antibiotic is a major contributor for their emergence [6]. VIM and NDM type of MBLs are horizontally transferable and found to be associated with mobile genetic elements, however few molecular information and genetic context is available from this part of India. The potential for quick and extensive dissemination rate of these resistance genes make over a great concern. The present study describes genetic background, transmission dynamics as well as stability of *bla*_VIM-2_ and *bla*_NDM-1_ in clinical isolates of *P. aeruginosa*.

## Methods

### Bacterial collection

From October 2012 to September 2013, a total of seventeen *P. aeruginosa* with reduced susceptibility to at least a single carbapenem were collected from Silchar Medical College and Hospital, Silchar. The isolates were then identified by standard biochemical reactions, cytochrome oxidase activity, citrate utilization, pigment production and growth on Cetrimide agar [7].

### Characterization of carbapenemases

For detection of carbapenemase production, isolates were subjected to modified Hodge test (MHT) and imipenem-EDTA disc test for metallo-β-lactamase production [4]. MHT uses *Escherichia coli* ATCC 25922 as an indicator organism. The presence of clover leaf like indentation in MHT was interpreted as a positive result for carbapenem hydrolysis.

### Molecular characterization of *bla*_VIM_ and *bla*_NDM_ gene

PCR assay was performed for detection of *bla*_VIM_ as described earlier [8] as well as other metallo-β-lactamase gene (*bla*_NDM_, *bla*_IMP_, *bla*_GIM_, *bla*_SIM_, *bla*_SMB_) [4] and amplified products were further sequenced to confirm the presence of resistant gene.

### Detection of co-existing of ESBL genes

Co-existence of ESBL genes were determined by multiplex PCR targeting *bla*_TEM_, *bla*_PER_, *bla*_OXA-2_, *bla*_SHV_, *bla*_CTX-M_, *bla*_VEB_ and *bla*_GES_ [9]. Reactions were performed under the following conditions: initial denaturation at 94 °C for 5 min, 33 cycles of 94 °C for 35 s, 51 °C for 1 min, 72 °C for 1 min and final extension at 72 °C for 7 min. The amplified products were further sequenced to confirm the co-existence of ESBL genes.

### Detection of genetic context of *bla*_VIM-2_

Genetic environment of *bla*_VIM-2_ was determined by performing integrase gene PCR [10] for characterizing the class 1 and class 2 integron. The conserved sequences 5′CS and 3′CS flanking the *bla*_VIM_ were determined using two sets of primers 5′CS and the reverse primer of *bla*_VIM_, 3′CS and forward primer of *bla*_VIM_ as described earlier [10]. Amplified products were sequenced to determine genetic map of *bla*_VIM-2_. The reaction condition was: initial denaturation at 95 °C for 3 min, 34 cycles at 95 °C for 30 s, 46 °C for 1 min, 72 °C for 3 min and final extension at 72 °C for 7 min. The presence of mobile element like ISCR in each strain was determined by performing PCR using primers ISCR F (5′- RNSBATAGGAADWWNAAHMNV-3′) and ISCR R (5′-BNKDTTNWWHTTCCTATVSNY-3′).

### Determination of genetic environment of *bla*_NDM-1_

Integron carriage was assessed by performing integrase gene PCR for characterizing the class 1 and class 2 integron. PCR reaction conditions followed was described as earlier [10]. The flanking region of *bla*_NDM_ were determined using two sets of primers 5CS and the reverse primer of *bla*_NDM_, 3CS and forward primer of *bla*_NDM_ [10]. The linkage of *bla*_NDM-1_ with insertion sequence IS*Aba125* was determined by using forward primer of IS*Aba125* (5′GAA ACT GTC GCA CCT CAT GTT TG-3′) and reverse of *bla*_NDM-1_ (5′-GTA GTG CTC AGT GTC GGC AT-3′) [11].

### Plasmid analysis, transformation and conjugation assay

*bla*_VIM_ positive bacterial isolates were cultured in Luria–Bertani broth (Hi-Media, India) containing 0.25 μg/ml of imipenem. After overnight incubation, plasmids were extracted by using QIAprep Spin Miniprep Kit (Qiagen, Germany). Isolated plasmids were transformed into recipient strain *Escherichia coli* JM107 by heat shock method and transformants were selected on LB agar with 0.25μg/ml of imipenem. Conjugation experiment was carried out using *bla*_VIM-2_ and *bla*_NDM-1_ harboring transformants as donors and a streptomycin resistant *E. coli* recipient strain B (Genei, India), both the donor and reciepient cells were cultured in Luria–Bertani Broth (Hi-Media, India) till it reach an O.D. of 0.8–0.9 at A_600_. Cells were mixed at 1:5 donor-to-recipient ratios and transconjugants were selected on imipenem (0.25 μg/ml) and streptomycin (400 μg/ml) agar plates. Additionally conjugation experiment was also tried using *P. aeruginosa* harboring *bla*_NDM-1_ and *bla*_VIM-2_ as donor and *E. coli* strain B as recipient.

### Southern hybridization for detection of transferability

Southern blotting was performed on agarose gel by in-gel hybridization with the *bla*_VIM-2_ and *bla*_NDM-1_ probe labelled with DIG HIGH PRIME LABELING MIX (Roche, Germany) detection Kit. The digoxigenin-labeled *bla*_VIM-2_ and *bla*_NDM-1_ specific probe was prepared using primers VIM-F, VIM-R, NDM-F and NDM-R. Plasmid DNA from transformants and transconjugants was separated by PFGE (CHEF DR-III System, Bio-Rad; USA) and transferred to nylon membrane (Hybond N, UK) and then hybridised with prepared *bla*_VIM_ and *bla*_NDM_ specific probe. Detection was performed by using an NBT color detection Kit (Roche, Germany).

### PCR based replicon typing

The incompatibility type of the plasmids encoding *bla*_VIM-2_ and *bla*_NDM-1_ were characterized by PCR based replicon typing targeting 18 different replicon types such as FIA, FIB, FIC, HI1, HI2, I1/Iγ, L/M, N, P, W, T, A/C, K, B/O, X, Y, F and FIIA [12].

### Antibiotic susceptibility and minimum inhibitory concentration (MIC)

Antimicrobial susceptibility of parent strains as well as transformants was determined by Kirby-Bauer disc diffusion method against β-lactam and non-β-lactam antibiotics (Hi-Media, India). MIC was also determined by agar dilution method for the isolates and the transformants carrying P^VIM−2^ and P^NDM−1^ towards imipenem (Merck, France), meropenem (AstraZeneca, UK), cefepime, aztreonam (Aristo, India), amikacin (Zuche pharmaceuticals, India), gentamicin (Pharmakem, India), ciprofloxacin(Ranbaxy, India), piperacillin-tazobactam (Alkem, India) and polymixin-B (Samarth, India) and interpreted as per CLSI guidelines [13].

### Stability of *bla*_VIM-2_ and *bla*_NDM-1_

Stability of *bla*_VIM-2_ and *bla*_NDM-1_ gene was determined by serial passage of the isolates as well as of the transformants in 1:1000 ratios without antibiotic pressure [14]. After each passage the test isolates were subjected to phenotypic detection of MBL and further confirmed the presence of *bla*_VIM_ and *bla*_NDM_ by PCR assay.

### Pulsed field gel electrophoresis

*bla*_VIM_ and *bla*_NDM_ positive isolates were typed by pulsed field gel electrophoresis where chromosomal DNA was prepared in agarose blocks and digested with restriction enzyme *Xba*1 (Promega, USA). DNA fragments were separated with CHEF-DR III apparatus (Bio-Rad, USA) and the electrophoresis conditions used were for 24 h at 6 V/cm with pulse rate of 10–40 s as described previously [15]. Clonal relatedness within the isolates was determined by comparing the band patterns.

### Ethical approval

The work was approved by Institutional Ethical committee of Assam University, Silchar vide Reference Number: IEC/AUS/C/2014-001. The authors confirm that participants provided their written informed consent to participate in this study.

## Results

**Fig. 1:**
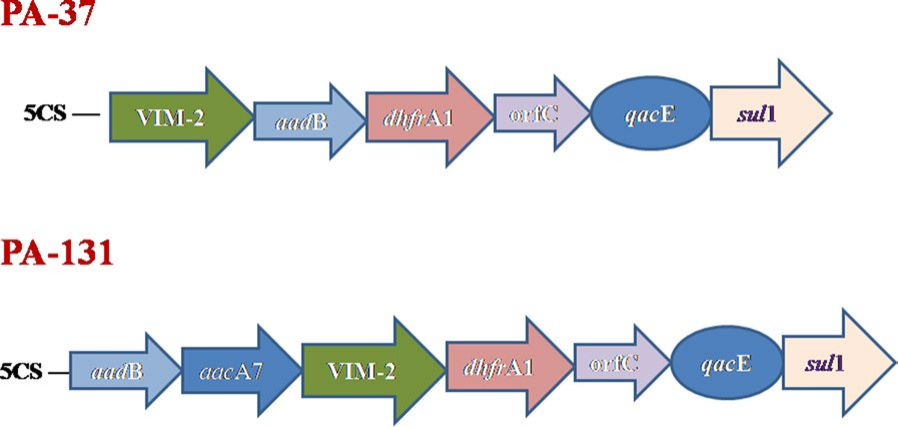
Genetic Context of *bla*_VIM-2_

**Fig. 2:**
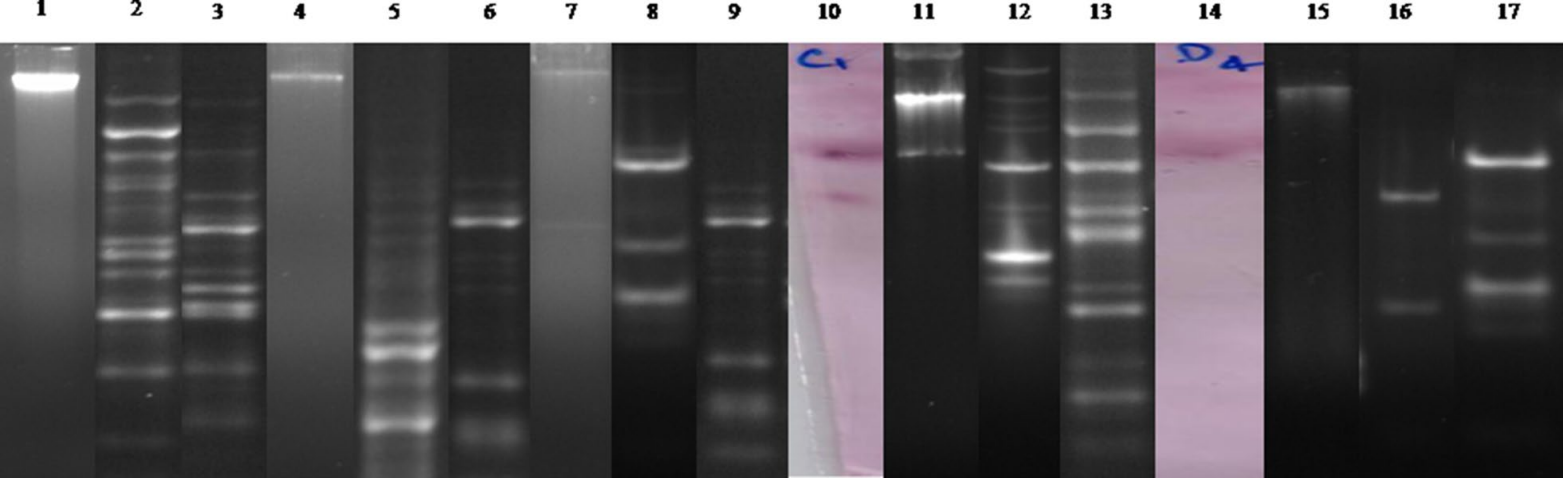
PFGE and hybridization analysis of *bla*_NDM-1_. *Lane 1* and *4* The undigested total DNA of PA 37 and PA 131 respectively; *Lane 2* and 3 The PA 37 total DNA is digested with S1 and *Xba* I respectively; *Lane 5* and *6* PA 131 total DNA digested with S1 and *Xba* I respectively; *Lane 7* The undigested plasmid of PA 37; *Lane 8* and *9* *E. coli* transconjugant carrying plasmid of PA 37 digested with S1 and *Xba* I respectively; *Lane 10* Hybridized P^PA 37^ with probe; *Lane 11* The undigested plasmid of PA 131; *Lane 12* and *13* *E. coli* transconjugant carrying plasmid of PA 131 digested with S1 and *Xba* I respectively; *Lane 14* Hybridized P^PA 131^ with probe; *Lane 15* The total DNA of recipient *E. coli* without plasmid; *Lane 16* and *17* The total DNA of recipient *E. coli* without plasmid digested with S1 and *Xba* I respectively

Two clinical isolates of *P. aeruginosa* (PA-37 and PA-131) harboring *bla*_VIM_ were recovered from Silchar Medical College and Hospital, India. The gene was further sequenced and confirmed as *bla*_VIM-2_ variant. The first isolate (PA-37) was recovered from pus samples of a 55 year old female patient suffering from wound infection admitted in surgery ward in December 2012 while the second one (PA-131) was isolated from urine of a 40 years old female patient with UTI, who attended gynecology outpatient department (OPD) in the month of February 2013. *bla*_VIM-2_ was found to be horizontally transferable as the gene could be successfully conjugatively transferred from transformed *E. coli* JM107 to recipient *E. coli* strain B through an Inc F type plasmid having approximate size of 30 kb. These findings were further confirmed by Southern hybridization results. However, conjugative transfer of plasmids from *P. aeruginosa* to *E. coli* was not successful with our experiment. In both the isolates *bla*_VIM-2_ was located within integron gene cassette and was flanked by other antimicrobial resistant determinant like gene for aminoglycoside resistance (Fig. 1). The two different types of cassette arrays observed were *bla*_VIM-2_-*aad*B-*dhfr*A-orfC-*qac*E-*sul*1 (PA-37) and *aad*B-*aac*A7-*bla*_VIM-2_-*dhfr*A1-orfC-*qac*E-sul1 (PA-131). Both the isolates were co-harboring *bla*_NDM-1_ and further ESBL screening revealed the presence of *bla*_VEB-1_ gene in these isolates. However, *bla*_NDM-1_ gene could not be hybridized with 30 kb plasmid that was harboring *bla*_VIM-2_, but with a 24 kb plasmid which was successfully hybridized with *bla*_NDM-1_ specific probe (Fig. 2). Linkage of IS*Aba125* was observed in the upstream region of *bla*_NDM-1_ whereas no association with integron gene cassette could be established. Interestingly on analyzing the stability of MBL genes i.e. *bla*_VIM-2_ and *bla*_NDM-1_, in case of wild type isolates these resistant genes were stable even after hundred consecutive passages but among their transformants, complete plasmid was lost after fifty-six passages in case of *bla*_VIM-2_ while *bla*_NDM-1_was retained till ninety passages without any antibiotic pressure. These *bla*_VIM-2_ positive isolates showed resistance towards most of the antibiotics including piperacillin/tazobactam, co-trimoxazole, amikacin, gentamicin, netilmicin and quinolone group of drugs. MIC values obtained for both the isolates were above breakpoint towards ciprofloxacin, aminoglycosides, β-lactam-β-lactamase inhibitor as well as to polymixin B (Table 1). The two *bla*_VIM-2_ harboring isolates of *P. aeruginosa* were found to be clonally different from each other on the basis of their PFGE banding pattern (Fig. 2).

**Table 1:** MIC (μg/ml) of the *bla*_VIM-2_ and *bla*_NDM-1_ harboring isolates and their transformants

Organisms	Imipenem	Meropenem	Cefepime	Aztreonam	Amikacin	Gentamicin	Ciprofloxacin	Piperacillin-tazobactam	Polymixin B
PA-37^a^	>256	>256	>256	>256	>256	>256	256	>256	>256
*E. coli* JM107 (p^VIM−2^/37)^b^	64	32	128	64	128	64	64	64	32
*E. coli* JM107 (p^NDM−1^/37)^c^	64	64	128	64	128	128	64	64	32
PA-131^a^	>256	128	>256	>256	>256	>256	256	>256	256
*E. coli* JM107 (p^VIM−2^/131)^b^	16	16	64	64	64	64	64	64	32
*E. coli* JM107 (p^NDM−1^/131)^c^	32	16	128	64	64	64	64	64	16
*E. coli* JM107	0.06	0.012	0.06	0.12	0.06	0.125	0.06	0.12	0.006

^a^Donor strain

^b^Recipient *E. coli* carrying plasmid of *bla*_VIM-2_

^c^Recipient *E. coli* carrying plasmid of *bla*_NDM-1_

## Discussion

It has been evidenced that the rapid emergence and dissemination of carbapenemase producing bacteria in this subcontinent is mainly due to the acquisition of *bla*_NDM_ [4]. But in the present study, we described an additional carbapenem resistance determinant i.e. *bla*_VIM-2_ which played a significant role in carbapenem resistance. Although *bla*_VIM-2_ with integron gene cassette is reported in previous studies [8], while in the present study, the genetic context of *bla*_VIM-2_ underscores their diverse origin and persistence along with other resistant genes. In the present study *bla*_VIM-2_ was found to be associated with the gene cassette along with other resistance determinants which is in agreement to the reports of Toleman et al. [16]. The association of other resistance determinants along with VIM type MBL confers the phenotype to become resistant to most of the available antimicrobial agents. The study reports the presence of aminoglycosides resistance genes *aad*B and *aac*A7 on the same gene cassette along with *bla*_VIM-2_, thus making the phenotype resistance to amikacin and gentamicin as well. In the year 2012, Toleman et al. [11] reported the association of insertion sequence IS*Aba125* in the upstream region of *bla*_NDM-1_ in *Acinetobacter baumanii*, we too observed the same insertion sequence present in the upstream region of *bla*_NDM-1_ in *P. aeruginosa*. Presence of NDM-1 in *P. aeruginosa* was for first time recorded in 2011 from patients in Serbia [17] and the same working group has reported that resistance determinant is chromosomally located in this particular organism [18]. Similarly, a report from India also has established its chromosomal location [19]. However, in our recent study we found presence of *bla*_NDM-1_ on plasmid DNA indicating a possible shift from one to another genetic location [20]. Future experiments will show which direction (chromosome to plasmid or vice versa) of the transfer took place in the organism. High MIC range as well as reduced susceptibility rate against majority of the tested antibiotics was observed in case of *bla*_VIM-2_ and *bla*_NDM-1_ harboring wild strains and transformants of *bla*_VIM-2_ and *bla*_NDM-1_. Earlier studies reported [16] that carbapenemase producing isolates remain susceptible to polymixin B whereas both of our study isolates were found to be resistant to this antibiotic, which could be a challenging situation with no or too limited treatment option. The association of mobile genetic element with *bla*_VIM-2_ may facilitate their mobilization to other susceptible organisms. On performing the transmission dynamics of the strains and it was evident that *bla*_VIM-2_ and *bla*_NDM-1_ was horizontally transferable. It may be noted that in our study, the plasmid of *P. aeruginosa* encoding the NDM-1 was conjugally transferred from the *E. coli* transformants to recipient *E. coli* strain although conjugation of the same plasmid was not successful from original host *P. aeruginosa* to *E. coli*. The reason for absence of plasmid conjugation could be due to presence of some physiological barriers within these two strains or we did not find the appropriate laboratory conditions for initiation of conjugation or present plasmid do not carry functional Tra operon in *P. aeruginosa*. Serial passage of transformants harboring both *bla*_VIM-2_ and *bla*_NDM-1_ showed that *bla*_NDM-1_ gene is more stable compare to *bla*_VIM-2_, may be because *E. coli* is an unnatural host for *bla*_VIM-2_. The distinguishable pulsotypes of the two strains along with their different genetic arrangements indicates horizontal acquisition from diverse source and antibiotic pressure in this hospital setting.

## Conclusions

Co-existence of multiple carbapenem resistance determinants in hospital isolate is worrisome and a matter of concern for infection control management considering the treatment option and clonal expansion. Thus, the current finding is of epidemiological interest, which requires immediate steps to initiate proper treatment option.

## Abbreviations

VIM: Verona integron-encoded MBL
NDM: New Delhi metallo-β-lactamase
MBLs: metallo-β-lactamases
*Bla*: beta-lactamase
MHT: modified Hodge test
PCR: polymerase chain reaction
SIM: Seoul imipenemase
GIM: German imipenemase
SMB: Serratia metallo-β-lactamase
ESBL: extended spectrum beta-lactamase
5CS & 3CS: 5′ & 3′-conserved segments
ISCR: insertion sequence common region
MIC: minimum inhibitory concentration
PFGE: pulse field gel electrophoresis
UTI: urinary tract infection
CLSI: Clinical and Laboratory Standards Institute
